# Supplementary material for: Callous–Unemotional Traits among Adolescents with Autism Spectrum Disorder, Attention-Deficit/Hyperactivity Disorder, or Typical Development: Differences between Adolescents’ and Parents’ Views
Source: Int J Environ Res Public Health. 2021 Apr 9;18(8):3972. doi: 10.3390/ijerph18083972 (PMC8069479; doi:10.3390/ijerph18083972)
Supplement: Supplementary file 1 [file ijerph-18-03972-s001.pdf]

**Supplementary Table S1.** Deomgraphics effect on Parent-Reported and Adolescent-Reported Callous–Unemotional Traits Among all groups.

| | Adolescent-reported | | | Parent-reported | | |
|---|---|---|---|---|---|---|
| | **Callousness** | **Uncaring** | **Unemotionality** | **Callousness** | **Uncaring** | **Unemotionality** |
| **Variable** | **B (SE)** | **B (SE)** | **B (SE)** | **B (SE)** | **B (SE)** | **B (SE)** |
| Age | -0.08 (0.11) | -0.15 (0.10) | 0.02 (0.06) | -0.17 (0.12) | -0.15 (0.11) | 0.21 (0.06) |
| Sex (girls vs. boys) | -1.64 (0.55)* | -1.04 (0.53)* | -0.24 (0.31) | -2.28 (0.64)*** | -1.66 (0.55)** | -0.43 (0.33) |

SE: standard deviation; *: $p < 0.05$; **: $p < 0.01$; ***: $p < 0.001$.

**Supplementary Table S2.** Adolescents' Sex and Age Distributions in TD, ADHD, and ASD Groups.

| | **TD** | | **ADHD** | | **ASD** | |
|---|---|---|---|---|---|---|
| | ***n* =126** | | ***n* =126** | | ***n* =126** | |
| **Variable** | **Mean (SD)** | ***n* (%)** | **Mean (SD)** | ***n* (%)** | **Mean (SD)** | ***n* (%)** |
| Age (years) | 13.88 (2.08) | | 13.12 (1.71) | | 13.66 (1.95) | |
| Sex | | | | | | |
| Girls | | 17 (13.5) | | 17 (13.5) | | 17 (13.5) |
| Boys | | 109 (86.5) | | 109 (86.5) | | 109 (86.5) |

ADHD: attention-deficit/hyperactivity disorder; ASD: autism spectrum disorder; SD: standard deviation; TD: typical development.

**Supplementary Table S3.** Comparisons of Parent-Reported and Adolescent-Reported Callous–Unemotional Traits Among TD, ADHD, and ASD Groups[a] .

| | **TD** | | **ADHD** | | **ASD** | | ***F*-value** | **Post-hoc comparison[b]** |
|---|---|---|---|---|---|---|---|---|
| | ***n* =126** | | ***n* =126** | | ***n* =126** | | | |
| **Variable** | **Mean** | **SD** | **Mean** | **SD** | **Mean** | **SD** | | |
| Adolescent-reported | | | | | | | | |
| Callousness | 6.70 | 4.03 | 9.42 | 5.02 | 9.89 | 5.46 | 15.75*** | ASD = ADHD > TD |
| Uncaring | 9.25 | 4.59 | 11.42 | 4.81 | 10.60 | 4.98 | 6.55** | ASD = ADHD > TD |
| Unemotionality | 6.90 | 2.48 | 7.35 | 2.84 | 7.56 | 2.94 | 1.91 | - |
| Parent-reported | | | | | | | | |
| Callousness | 8.10 | 4.69 | 12.11 | 5.58 | 12.94 | 5.65 | 29.81*** | ASD = ADHD > TD |
| Uncaring | 10.51 | 4.40 | 13.60 | 4.49 | 13.92 | 5.07 | 20.61*** | ASD = ADHD > TD |
| Unemotionality | 6.34 | 2.63 | 5.78 | 3.14 | 7.19 | 3.02 | 7.38*** | ASD > ADHD = TD |

[a] : ANOVA; [b]: Shaffer's correction; ADHD: attention-deficit/hyperactivity disorder; ASD: autism spectrum disorder; SD: standard deviation; TD: typical development; **: $p < 0.01$; ***: $p < 0.001$.

**Supplementary Table S4.** Comparisons of Difference Scores Between Parent-Reported and Adolescent-Reported Callous–Unemotional Traits Among TD, ADHD and ASD Groups [a].

| | **TD** | | **ADHD** | | **ASD** | | ***F*-value** | **Post-hoc comparison[b]** |
|---|---|---|---|---|---|---|---|---|
| | ***n* =126** | | ***n* =126** | | ***n* =126** | | | |
| **Variable** | **Mean** | **SD** | **Mean** | **SD** | **Mean** | **SD** | | |
| Callousness | 1.40 | 5.46 | 2.69 | 6.01 | 3.05 | 6.51 | 2.63 | - |
| Uncaring | 1.25 | 5.18 | 2.18 | 4.91 | 3.33 | 5.18 | 5.24** | ASD > TD |
| Unemotionality | -0.56 | 3.23 | -1.57 | 3.72 | -0.37 | 3.91 | 3.98* | ADHD > ASD |

[a] : ANOVA; [b]: Shaffer's correction; ADHD: attention-deficit/hyperactivity disorder; ASD: autism spectrum disorder; SD: standard deviation; TD: typical development; *: $p < 0.05$; **: $p < 0.01$.
